# Recombinant neuraminidase pseudotyped baculovirus: a dual vector for delivery of Angiotensin II peptides and DNA vaccine

**DOI:** 10.1186/s13568-018-0699-8

**Published:** 2018-10-16

**Authors:** Irisa Trianti, Saengchai Akeprathumchai, Phenjun Mekvichitsaeng, Sansanalak Rachdawong, Kanokwan Poomputsa

**Affiliations:** 1 Biotechnology Program, School of Bioresources and Technology, King Mongkut’s University of Technology Thonburi, 49 Soi Thian Thale 25, Bang Khun Thian Chai Thale Road, Tha Kham, Bang Khun Thian, Bangkok, 10150 Thailand; 2 Pilot Plant Development and Training Institute, King Mongkut’s University of Technology Thonburi, 49 Soi Thian Thale 25, Bang Khun Thian Chai Thale Road, Tha Kham, Bang Khun Thian, Bangkok, 10150 Thailand; 3 KMUTT Science and Industrial Park, King Mongkut’s University of Technology Thonburi, 49 Soi Thian Thale 25, Bang Khun Thian Chai Thale Road, Tha Kham, Bang Khun Thian, Bangkok, 10150 Thailand

**Keywords:** Pseudotyped baculovirus, Neuraminidase (NA), Angiotensin II (AngII), Hypertension, DNA vaccine, AngII peptide vaccine

## Abstract

Baculovirus is a promising vaccine deliver vector due to its biosafety profiles, gene transfer efficiency, ability to display small foreign antigens on its surface, strong adjuvant activities, etc. A dual vector for peptide antigens and a DNA vaccine delivery was constructed. In this vector, a tetrameric glycoprotein neuraminidase (NA) from influenza A virus (H5N1) serves as a baculovirus surface protein to improve baculovirus transduction efficiency and a partner for displaying the target peptide antigen. Nucleotides encoding target peptides could be fused to a full length NA gene, at the lower part of its head structure, integrated into *Autographa californica* multinucleopolyhedrovirus genome and expressed under the control of a White Spot Syndrome Virus IE-1 shuttle promoter. Angiotensin II (AngII) peptides, a potent vasoconstrictor that causes high blood pressure, was our target antigen. The recombinant NA-AngII pseudotyped baculovirus had the AngII peptides fused to the NA and displayed on its surface. In vitro studies revealed that this recombinant baculovirus successfully delivered AngII peptides, as DNA vaccine, into human HEK293A cells. A single subcutaneous injection of the recombinant NA-AngII pseudotyped baculovirus into moderately high blood pressure rats at 4 × 10^9^ pfu/rat, stimulated anti-AngII antibody production and their systolic blood pressure (SBP) levels were found to have decreased. In addition, a single intranasal immunization at 8 × 10^8^ pfu/rat, raised anti-AngII antibodies in a rat and its SBP was also reduced. The recombinant neuraminidase pseudotyped baculovirus is a potential vector for AngII peptide antigen and DNA vaccine for subcutaneous or intranasal immunization for treatment of hypertension.

## Introduction

The insect baculovirus *Autographa californica* multinucleopolyhedrovirus (AcMNPV) is an enveloped virus containing a circular double-stranded DNA genome with ease of genetic manipulation and high titer production (Airenne et al. 2013). When equipped with an appropriate eukaryotic promoter, baculovirus has high transduction efficiency in many cell types including mammalian and human cells with low cytotoxicity (Hofmann et al. 1995; Blom et al. 2003; Gao et al. 2007). Small target molecules can be displayed on the baculovirus surface by fusion with baculovirus surface proteins such as AcMNPV major glycoprotein gp64 that locates on the baculovirus head domain (Grabherr et al. 1997; Ernest et al. 1998). Other foreign transmembrane proteins such as influenza virus neuraminidase (NA) and vesicular stomatitis virus G protein (VSV-G) were also employed to display peptides on the baculovirus lateral regions (Chapple and Jones 2002; Borg et al. 2004). Baculovirus is known for its adjuvant properties by enhancing humoral and cellular immune responses against target antigens. In addition, baculovirus activates innate immune responses by inducing type I and II IFNs (Abe et al. 2005; Hervas-Stubbs et al. 2007; Suzuki et al. 2010; Heinmäki et al. 2017) and has ability to stimulate mucosal immune system by a needle-free, painless and stress free administration. Intranasal administrations with the recombinant hemagglutinin (HA) baculovirus were reported to protect immunized mice from a lethal influenza infection (Abe et al. 2003). In addition, oral administration of recombinant baculovirus expressing H5N1 vaccine induced high level of mucosal, and systemic immune responses (Prabakaran et al. 2010).

A recombinant neuraminidase pseudotyped baculovirus was designed and constructed for delivery of Angiotensin II (AngII) peptide antigen and AngII DNA vaccine. AngII is an active peptides in renin angiotensin system (RAS). Binding of the AngII peptides to its receptor causes vasoconstriction and induces aldosterone secretion, leading to sodium resorption in the kidney and hence raising blood pressure (Oparil et al. 2003). It has been well documented that AngII conjugated with carriers such as virus like particles (CTY006-AngQb) effectively induced anti-AngII antibodies that led to significantly reduced blood pressure (Tissot et al. 2008). In addition, DNA vaccine from AngII DNA fusion with hepatitis B core (HBc) protein significantly lowered the blood pressure in spontaneous hypertension rats (SHR) for at least 6 months (Korimaya et al. 2015). AngII peptides are therefore an excellent vaccine antigen for hypertension therapy in both peptides and DNA forms (Pandey et al. 2009). In this study, a homo-tetrameric surface glycoprotein neuraminidase from influenza A virus (H5N1), with an enzymatic head domain and major antigenic sites connected by a stalk to an N-terminal transmembrane domain, was chosen to serve as baculovirus pseudotyped surface protein and fusion partner for displaying the AngII peptides. NA fusion with a foreign epitope was previously reported by Castrucci et al. (1992) in which the NA amino acids from residues 63 to 70, according to the NA of the influenza A/WSN/33 (H1N1) numbering, were substituted by a FLAG peptide at the lower part of the head structure. It was found that FLAG was on an exposed location on the influenza virion surface. Since the recombinant neuraminidase pseudotyped baculovirus had been surface modified, it should be able to evade complement-mediated destruction as has been shown with VSV-G pseudotyped baculoviruses (Barsoum et al. 1997; Tani et al. 2001; Pidre et al. 2013). NA contains secondary sialic acid binding sites which are not in the active site (Laver et al. 1984) that may play some roles in facilitating binding of the recombinant neuraminidase pseudotyped baculovirus to sialic acid receptors on the host cell (Votapka et al. 2012). The NA-AngII fusion gene was designed to be expressed under the control of a White Spot Syndrome Virus (WSSV) IE-1 shuttle promoter which is active in both insect and mammalian cells. Thus, the recombinant neuraminidase pseudotyped baculovirus is expected to have high transduction efficiency and ability to deliver two types of antigens i.e. peptide antigen produced by insect cells as a part of baculovirus particles and DNA carried by baculovirus genome for expression in target mammalian cells.

## Materials and methods

### Plasmid and bacterial strain

pFastBac-IE1 was modified from pFastBac™HT B, a donor plasmid used for the construction of recombinant baculovirus in the Bac-to-Bac^®^ Baculovirus Expression System (Invitrogen, USA) (Trianti et al. 2016). The original baculovirus polyhedrin promoter was replaced with WSSV immediate early-1 (IE-1) promoter. DH10Bac™competent *E. coli* cells contain helper plasmid and a modified baculovirus genome (bacmid, Invitrogen, USA).

### Cells

*Spodoptera frugiperda* insect cells (Sf-9, ATCC CRL-1711) were maintained as a suspension culture using TMN-FH medium (Invitrogen, USA) supplemented with 10% heat inactivated Fetal Bovine Serum, FBS (Hyclone, USA) and incubated at 27 °C. Human Embryonic Kidney 293A cells (HEK293A, Invitrogen, USA) were cultured in DMEM medium (Gibco, USA) with 10% FBS at 37 °C.

### Generation of recombinant neuraminidase pseudotyped baculovirus displaying AngII peptides

The influenza neuraminidase pseudotyped baculovirus displaying AngII peptides (rBV-NA-AngII), was generated as described by Trianti et al. (2016). Briefly, the Influenza virus A/Thailand/1(KAN1)/2004 (H5N1) neuraminidase gene (GenBank no. AY555151, Puthavathana et al. 2005) kindly provided by Professor Pilaipan Puthavathana, Faculty of Medicine Siriraj Hospital, Mahidol University, Thailand, was fused with nucleotides encoding Angiotensin II (AngII) peptides by overlapping extension PCR (Hoa et al. 1989). The NA-AngII fusion PCR product with *Rsr*II and *Not*I restriction enzymes at 5′ end and 3′ end, respectively, was ligated into a donor plasmid, pFastBac-IE1, at *Rsr*II and *Not*I cloning sites downstream of the WSSV IE-1 shuttle promoter. The recombinant donor plasmid was then transformed into DH10Bac™ *E. coli* where the NA-AngII fusion gene transposed into the baculovirus genome (bacmid) through site-specific transposition as described by the manufacturer. This recombinant bacmid was then extracted from transformed DH10Bac™ *E. coli* and transfected into 80% confluent Sf-9 cells with Cellfectin II (Invitrogen, USA). Transfected Sf-9 cells were incubated at 27 °C for 72 h and the culture medium containing the budded form of recombinant baculovirus was harvested. The recombinant NA-AngII pseudotyped baculovirus was further amplified by infection into Sf-9 insect cells using multiplicity of infection (MOI) of 0.1 for 5 days and their titer determined by end-point dilution assay (O’Reilly et al. 1992). Virus was concentrated by ultracentrifugation at 24,000 rpm in a SW 28 rotor using an optima L-100XP ultracentrifuge (Beckman Coulter, USA). The virus pellet was resuspended in sterile PBS and stored at 4 °C before use.

### Gene expression analysis

NA-AngII fusion gene expression analysis was carried out by reverse transcription-PCR (RT-PCR). Briefly, the transfected cells were harvested and extracted by addition of TRIZOL (Invitrogen, USA) and their total RNA was obtained. The first-strand cDNA synthesis reaction was catalyzed by Reverse Transcriptase following the manufacturers (Thermo scientific, USA) using oligo (dT) primers. The synthesized cDNA was then used as template for PCR using primers specific to NA sequences at 5′ and 3′ for detection of a full length NA-AngII transcript and the NA specific forward primer (5′ CGCCGGTCCGAAACCATGAATCCAAATAAGAAG 3′) and AngII specific reverse primer (5′ GAGGCTGAAGGG GTGTATGTACACCCG GTC 3′) for detection of an AngII transcript.

### Western blot analysis

The culture supernatant collected from the rBV-NA-AngII infected Sf-9 cells was subjected to 12% SDS-PAGE and transferred to a nitrocellulose membrane (Bio-Rad, USA). For NA detection on the baculovirus, anti-NA polyclonal antibody (GeneTex, USA), at a dilution of 1:500, was used as a primary antibody and the goat anti-rabbit IgG conjugated with horseradish peroxidase (HRP) (Cell Signaling Technology, USA), at dilution of 1:2000, was used as a secondary antibody. The anti-NA antibody specific protein band was developed by 3, 3′, 5, 5′-Tetramethylbenzidine (TMB) liquid substrate (Sigma, USA).

For detection of AngII peptides on the baculovirus, anti-AngII monoclonal antibody (Abcam, UK) at a dilution of 1:500 was used as a primary antibody and the goat anti-mouse IgG conjugated with alkaline phosphatase (AP) (Sigma, USA) at dilution of 1:2000 was used as a secondary antibody. Anti-AngII antibody specific protein bands were developed using AP conjugate substrate (Bio-Rad, USA). Culture supernatant of Sf-9 cells infected with AcMNPV baculovirus, from bacmid DNA transfection, was used as negative control for both Western blots.

### Immuno-gold labeling and electron microscope analysis

Immune-gold labelling of the rBV-NA-AngII was performed as previously described by Yoshida et al. (2010) with some modifications. Briefly, formvar carbon coated grids (EMS, USA) were floated on 10 µl of recombinant baculovirus suspension for 30 min, before being floated onto anti-AngII monoclonal antibody (Abcam, UK) for 1 h. After washing three times with PBS, the grid was floated on anti-mouse IgG conjugated with 5 nm gold particles (Sigma, USA) for 30 min followed by washing three times with PBS. Grids were stained with 2% phosphotungstic acid (pH 7.2) for 10 min (Sigma, USA), air dried for 20 min and examined using a transmission electron microscope HT7700 (Hitachi, Japan).

### Rat immunization and blood pressure monitoring

Five sixteen-week old male Wistar rats (weight, 240–250 grams) were used. Two rats were subcutaneously injected with 4 × 10^9^ pfu/rat of the rBV-NA-AngII emulsified in complete Freund’s adjuvant in 500 μl. PBS at the same volume was used as a negative control. For intranasal immunization, two rats were given nasal drops with 8 × 10^8^ pfu/rat of rBV-NA-AngII, without adjuvant, in two drops (25 µl/drop) at 3 min interval. Systolic blood pressure of all immunized rats was monitored by tail cuff method (Kent scientific, USA) using coda system before immunization and at day 5 and 7 post immunization. Seven days post-immunization, immunized rat sera were collected from heart for detection of specific antibodies against the recombinant NA-AngII fusion protein by Western blot analysis.

### Mammalian cell transduction

HEK293A cells, cultured in DMEM medium supplemented with 10% FBS, were seeded into 6-well-plates at 3 × 10^5^ cells/well and incubated at 37 °C overnight. Cells were washed three times with PBS and the rBV-NA-AngII at MOI of 6 was added and cells incubated at 27 °C. Wild type AcMNPV baculovirus at the same MOI was used as a negative control. Three hours post-transduction, baculovirus suspensions were replaced with fresh DMEM medium supplemented with 10% FBS and incubated at 37 °C for 48 h. Transduced cells were collected, washed 3 times with PBS and cell lysates prepared in RIPA buffer for detection of recombinant NA-AngII fusion protein by Western blot analysis.

## Results

### Recombinant neuraminidase pseudotyped baculovirus as AngII peptide presenting vector

**Fig. 1:**
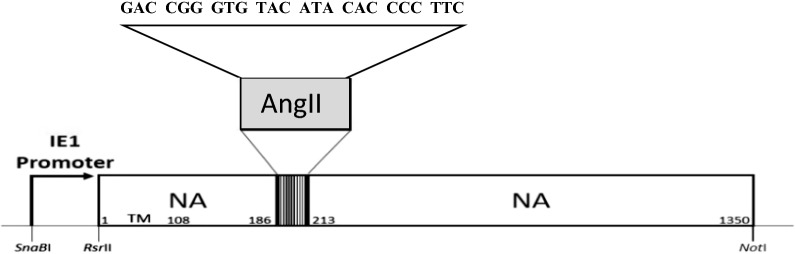
An NA-AngII fusion gene under the control of WSSV IE1 promoter. The NA amino acids from residues 63 to 70, according to the NA of the influenza A/WSN/33 (H1N1) numbering, and corresponding to nt 187 to nt 210, were substituted by an AngII peptide encoding nucleotides (GAC CGG GTG TAC ATA CAC CCC TTC)

**Fig. 2:**
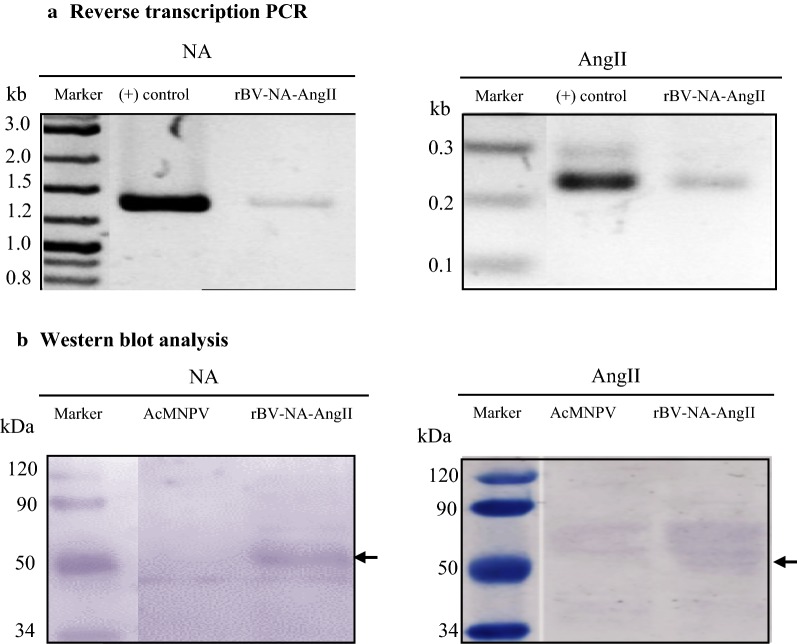
NA and AngII fusion gene expression and recombinant NA-AngII fusion protein analysis in the rBV-NA-AngII infected Sf9 insect cells by reverse transcription-PCR (**a**) and Western blot analysis (**b**), respectively. For reverse-transcription PCR, primers specific to neuraminidase (NA) and AngII peptides nucleotide sequences (AngII) were used for PCR of cDNA prepared from rBA-NA-AngII infected Sf-9 cells. A plasmid with NA-AngII fusion gene was used as a (+) control for PCR. For Western blot analysis, an anti-NA polyclonal antibody for detection of recombinant neuraminidase protein and an anti-AngII monoclonal antibody for detection of AngII peptides (AngII), as a part of the recombinant neuraminidase protein, were used. Sf9 insect cells infected with wild type AcMNPV was used as a (−) control. Arrow indicates recombinant NA-AngII fusion protein

**Fig. 3:**
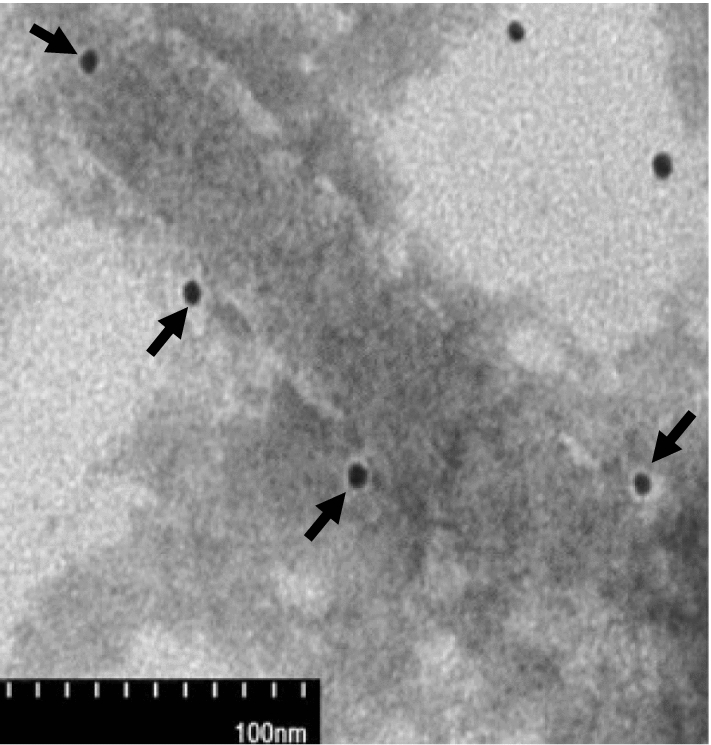
Electron micrograph of recombinant NA pseudotyped baculovirus displaying AngII peptides showing NA-AngII fusion protein on baculovirus surface. The displayed AngII peptides were labelled with anti-AngII monoclonal antibody as a primary antibody and anti-mouse IgG conjugated with 5 nm gold as a secondary antibody. Arrows indicate gold particles

AcMNPV baculovirus was pseudotyped with influenza A (H5N1) neuraminidase fused with an AngII peptide. The NA-AngII fusion gene fragment was integrated into the baculovirus genome (bacmid) and expressed under the control of WSSV IE-1 promoter (Trianti et al. 2016), (Fig. 1). Gene expression was confirmed by RT-PCR in which the AngII encoding nucleotides were found within the NA transcript (Fig. 2a). Western blot analysis of the recombinant baculovirus revealed a protein being recognized by both anti-NA polyclonal antibody and anti-AngII peptide monoclonal antibody with a molecular weight of approximately 55 kDa which corresponded to the neuraminidase-AngII fusion protein (Fig. 2b). Translocation of this AngII peptide on the modified baculovirus, namely rBV-NA-AngII, was revealed by immuno-gold electron microscopy. Anti-AngII monoclonal antibodies specifically bound to the surface of the pseudotyped baculovirus as shown by distribution of gold particles conjugated with anti-mouse secondary antibody (Fig. 3) indicating that the AngII peptides were exposed on the surface of the recombinant neuraminidase-AngII pseudotyped baculovirus.

### Recombinant neuraminidase-AngII pseudotyped baculovirus as a DNA vaccine deliver vector

**Fig. 4:**
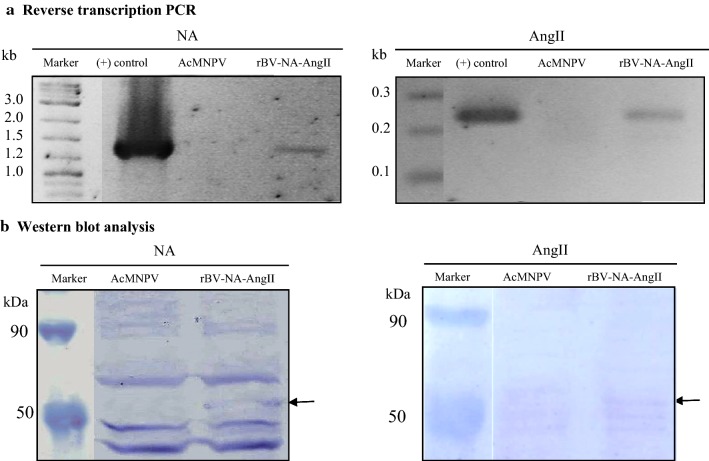
NA and AngII fusion gene expression and recombinant NA-AngII fusion protein production analysis in rBV-NA-AngII transduced HEK293 cells by reverse-transcription PCR (**a**) and Western blot analysis (**b**), respectively. cDNA and proteins obtained from a wild type AcMNPV transduced HEK293 cells were (−) controls for **a** and **b**, respectively. For reverse transcription PCR, primers specific to neuraminidase and AngII peptide nucleotide sequences were used for PCR of cDNA prepared from the rBA-NA-AngII transduced HEK293 cells. A plasmid with the NA-AngII fusion gene was a (+) control. For Western blot analysis, an anti-NA polyclonal antibody for detection of recombinant neuraminidase protein and an anti-AngII monoclonal antibody for detection of AngII peptides, as a part of the recombinant neuraminidase protein, were used to detect recombinant NA-AngII fusion protein. Arrow indicates recombinant NA-AngII fusion protein

Recombinant neuraminidase-AngII pseudotyped baculovirus had been shown to successfully display AngII peptides on its surface for effectively presenting these peptide antigens to the immune system. This rBV-NA-AngII baculovirus had been designed (Trianti et al. 2016) for expression of the NA-AngII fusion gene as a DNA vaccine in mammalian cells using the WSSV IE-1 shuttle promoter. NA-AngII gene expression after transduction of the rBV-NA-AngII baculovirus into human embryonic kidney cell line, HEK293A, was confirmed. Reverse Transcription-PCR of total RNA from transduced HEK293A cell lysates produced specific mRNA transcripts at the expected sizes of both the full length NA-AngII fusion gene and half NA-AngII indicating the presence of the nucleotides coding for AngII peptides (Fig. 4a). For protein synthesis, Western blot analysis of the cell lysates obtained from transduced HEK293A cells revealed a 55 kDa recombinant protein specifically bound to both anti-NA polyclonal antibody and anti-AngII monoclonal antibody (Fig. 4b). This protein was not detected in the wild type AcMNPV baculovirus transduced HEK293A cell lysate. These in vitro assays suggested that the rBV-NA-AngII could be used as an AngII DNA vaccine delivery vector in mammalian cells as both AngII mRNA and peptides were synthesized by the transduced cells.

### Rat immune response to recombinant neuraminidase-AngII pseudotyped baculovirus

**Fig. 5:**
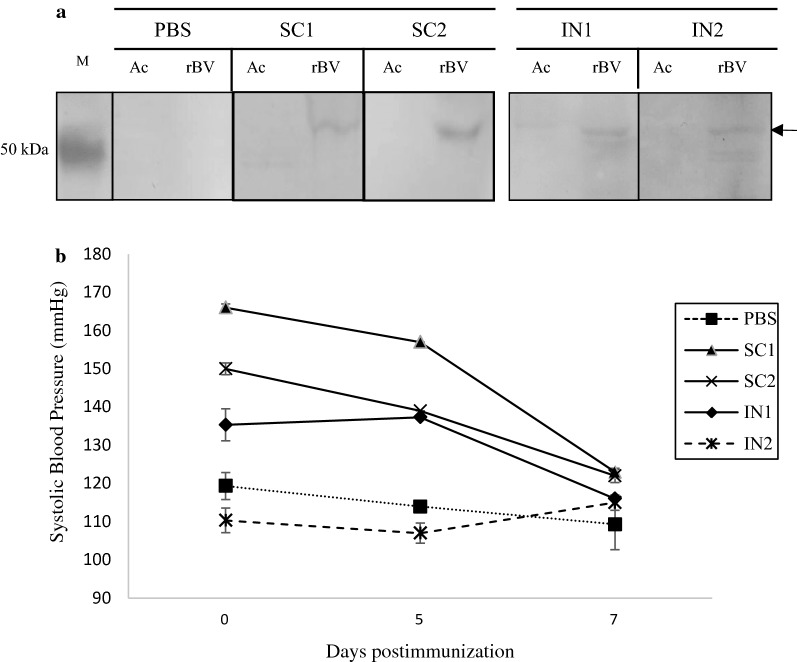
Rat immune responses to the rBV-NA-AngII after subcutaneous injection (SC) and intranasal administration (IN). SC injection with PBS was a (-) control. Antibodies in immunized rat sera were detected by binding of the antibodies to the rBV-NA-AngII (rBV) on the Western blot compared with the wild type AcMNPV (Ac), arrow indicates recombinant NA-AngII fusion protein (**a**). Systolic blood pressure (SBP) of immunized rats on day 0, 5 and 7 post immunization (**b**)

Immunogenicity of the recombinant neuraminidase-AngII pseudotyped baculovirus was assessed. Two rats were subcutaneously injected (SC) with rBV-NA-AngII at 4 × 10^9^ pfu/rat. Rat sera were collected after 7 days post immunization, and subjected to Western blot analysis. Figure 5a shows both rat sera contained some antibodies which are specific to a band at 55 kDa of recombinant NA-AngII protein. These antibodies did not bind to any proteins of wild type AcMNPV baculovirus. Similar results were found in sera collected from rats which had been treated by intranasal immunization (IN) with the rBV-NA-AngII baculovirus at 4 × 10^8^ pfu/rat, 5 times less virus than used for IC (Fig. 5a). This suggested the rBV-NA-AngII treated rat could be immunized by subcutaneous injection as well as nasal drop to induce antibodies specific to the recombinant NA-AngII fusion protein.

### Effect of anti-AngII antibody on rat systolic blood pressure

Systolic blood pressure (SBP) levels of immunized rats were monitored on day 0, 5 and 7. SBPs of two rats which had been subcutaneously immunized (SC) with the rBV-NA-AngII reduced over time (Fig. 5b) while the SBP of a control rat immunized with PBS showed no significant change. These results suggested the presence of antibodies that were able to neutralize serum AngII peptides, anti-AngII antibodies, in their sera. For intranasal (IN) immunized rats, only the rat with a slightly high SBP on day 0, had SBP levels reduced. This was probably due to the anti-AngII antibody that had been induced by the immunization as described for both SC immunized rats. The other intranasal immunized rat which had quite low SBPs showed no reduction in its SBP levels over a 7 days periods.

## Discussion

A recombinant baculovirus was constructed as a dual vector for peptide antigen and DNA vaccine delivery. An influenza virus surface protein, neuraminidase (NA) from Influenza A virus (A/Thailand/Kan353/2004(H5N1)) was employed for pseudotyping the baculovirus for two purposes. Firstly, to increase baculovirus transduction efficiency into target cells, as previously described. Secondly, to take advantage of the NA structure for peptide display by fusion of the peptides of interest into the lower part of the NA head structure. Nucleotides encoding AngII peptides, our target antigen, were fused into the lower part of NA head structure under the control of WSSV IE-1 promoter. After introduction of the NA-AngII fusion gene into the baculovirus genome, the resultant recombinant baculovirus, rBV-NA-AngII was efficiently produced from Sf9 insect cells in high titers, ranging from 4 × 10^8^ to 4 × 10^9^ pfu/ml. Thus, baculovirus surface modifications with the NA-AngII fusion protein did not compromise its replication process (Fig. 2b). The recombinant NA-AngII fusion protein was found not only localized on the baculovirus pole structure but also the lateral region of the virion surface (Fig. 3) similar to the VSV-G pseudotyped protein (Chapple and Jones 2002). AngII peptides should be displayed on each NA monomer and hence four peptides/NA molecules could be expected (Castrucci et al. 1992; Saito et al. 1995). The beneficial structure of the rBV-NA-AngII in enrichment of AngII antigen offers greater immune stimulation for anti-AngII antibody production. Furthermore, it had been previously reported that host-synthesized antigens from a DNA vaccine is capable of eliciting immune responses via both MHC‐I and MHC‐II pathways (Donnelly et al. 1997). The rBV-NA-AngII genome carried the NA-AngII fusion gene under the control of the WSSV-IE1 promoter. Upon transduction into the HEK293A cells, the gene was expressed (Fig. 4a) and the recombinant NA-AngII fusion protein synthesized as detected by Western blot analysis (Fig. 4b). Dual forms of antigens, AngII peptides and DNA vaccine delivered by the rBV-NA-AngII, were made possible by the WSSV IE-1 promoter which is active in both insect cells and HEK293A cells. WSSV IE-1 has been found to induce higher level of humoral and cellular immunity compared to CMV promoter (Ge et al. 2016).

Immune stimulation efficacy of the rBV-NA-AngII was investigated in rats. Seven days after a single subcutaneous injection of this baculovirus into two moderately high blood pressure rats, antibodies specific to the 55 kDa recombinant NA-AngII protein were detected in their sera (Fig. 5a). This specific signal possibly from the binding of anti-NA protein antibody or anti-AngII antibody or both antibodies to the recombinant NA-AngII protein. Decreasing levels of systolic blood pressure (SBP) in both rats indicated the presence of anti-AngII antibody in the immunized rat sera. These anti-AngII antibodies bound to the serum AngII, blocked the RAAS system and subsequently lowered the rat SBP levels (Fig. 5b). Induction of AngII peptide specific antibodies, after only a single immunization in this study, could be due to (i) strong adjuvant activities of the surface modified baculovirus (ii) DNA priming immunization by the NA-AngII fusion gene delivered into the nucleus of immune cells which could induce high-level antigen specific B cell response (Li et al. 2014) and (iii) AngII peptides on the NA structure and baculovirus surface being well displayed and recognized by the immune cells.

Baculovirus has been reported to activate mucosal immunity through DCs and macrophages which are found abundantly in a mucosal inductive site in the upper respiratory tract, through a TLR9-dependent pathway (Zuercher et al. 2002; Abe et al. 2003, 2005). In this study, the rBV-NA-AngII was also intranasal administered into two rats, at 5 times lower (8 × 10^8^ pfu/ml) than subcutaneously injected rats (4 × 10^9^ pfu/ml). Only the immunized rat which had a slightly higher SBP showed a decrease in SBP level while the rat with normal SBP levels showed no change (Fig. 5a). This finding was similar to a previous report by Nakagami et al. (2013) in which no BP level was changed in normal SBP mice by anti-Ang II antibody. They, and others, also confirmed that eight amino acids of Ang II could activate B cells but not T cells in an animal model. Thus, Ang II antigen is safe due to no risk of anti-Ang II T cell-mediated autoimmune response, and effectively induces the production of anti-Ang II antibody.

Antibodies specific to the recombinant NA-AngII protein were detected in IN immunized rat sera on day 7 post immunization (Fig. 5a). These results corresponded with the nasal drop immunization with an AcMNPV-based PyMSP119, malaria parasite antigen which induced strong systemic humoral immune responses (Yoshida et al. 2010). Similarly, intranasal immunization of live baculovirus display influenza virus (H7N7) hemagglutinin (HA) also induced antibody production, both IgG and IgA, after the first immunization (Rajesh Kumar et al. 2013). In addition to general baculovirus immune induction properties, neuraminidase may play a role in helping the rBV-NA-AngII to move through the mucus by degradation of mucin and extracellular glycolax (Matrosvich et al. 2004) or facilitate virus entry to cells via receptor mediated endocytosis (Ohuchi et al. 2006). We also observed neuraminidase activity from the concentrated rBV-NA-AngII sample (data not shown) which could as well facilitate virus entry to the cells. More studies on dose effect of the rBV-NA-AngII, pharmacokinetics and further improvements of this baculovirus such as removal of bacmid selectable markers, bypassing complement mediated inactivation, etc. are recommended.

In this study, the rBV-NA-AngII has been demonstrated as a promising dual vector for AngII peptide antigen and AngII DNA vaccines. This baculovirus vector could be modified to be used with other peptides and DNA vaccine antigens. The baculovirus could be pseudotyped by the NA-X fusion protein, where X is a target peptide antigen encoding nucleotides inserted into the NA at the bottom of its head portion. By using the WSSV-IE1 promoter, not only the target peptide antigen will be displayed on each NA, but also allows NA-X gene expression in target animals or humans. Another promising feature of this vaccine model is the ability to raise the immune system via the intranasal route without the need for an adjuvant. The intranasal route is “needle free” and can be frequently used by the patient thus making it suitable for therapeutic vaccines.

## Abbreviations

AcMNPV: *Autographa californica* multinucleopolyhedrovirus
AngII: angiotensin II
IE-1: immediate early-1
FBS: fetal bovine serum
IN: intranasal
MOI: multiplicity of infection
NA: neuraminidase
WSSV: White Spot Syndrome Virus
pfu: plaque forming unit
RAS: renin angiotensin system
SBP: systolic blood pressure
rBV-NA-AngII: recombinant NA-AngII pseudotyped baculovirus
SC: subcutaneous
TMB: 3, 3′, 5, 5′ Tetramethylbenzidine
VSVG: vesicular stomatitis virus G protein
